# Ocular drift shakes the stationary view on pattern vision

**DOI:** 10.1167/jov.25.8.17

**Published:** 2025-07-23

**Authors:** Lynn Schmittwilken, Marianne Maertens

**Affiliations:** 1 Computational Psychology, Electrical Engineering and Computer Science Technische Universität, Berlin, Germany

**Keywords:** edge sensitivity, contrast sensitivity, active vision, spatial vision, fixational eye movements

## Abstract

The mechanisms by which the visual system extracts key features (i.e., edges) from the visual input remain not fully understood. As reflected in the term *spatial vision*, pattern vision is traditionally assumed to operate on stationary visual inputs. However, our eyes are never truly still. Involuntary eye movements, specifically ocular drift, continuously alter the visual input during fixations and redistribute its power, emphasizing high spatial frequency contents. In this study, we examine the role of ocular drift on edge sensitivity in noise. We show that drift-induced shifts in stimulus power lead to better predictions of the empirical data, consistent with the human contrast sensitivity function. We then incorporate drift into a mechanistic model of spatial vision to test whether this further improves model predictions. Surprisingly, the original spatial model outperforms the drift-enhanced version. It does so in an interesting way: It artificially compensates for the absence of drift by redistributing the activity across its spatial frequency channels in later processing stages, effectively mimicking the effect of a dynamic input without explicitly modeling it. By contrast, a simpler model with a single spatial frequency channel benefits from drift but performs poorly when drift is removed. These findings suggest that standard model architectures inherently favor a stationary view of visual processing, which could result in self-confirming theories. Incorporating the dynamic nature of the visual input may offer a more accurate model of how the brain processes key features of natural scenes. However, doing so requires a critical reassessment of long-standing frameworks in visual neuroscience.

## Introduction

Edges are luminance (or texture) discontinuities in two-dimensional (2D) images, which often signal the boundaries of objects in the three-dimensional (3D) world. Edges are essential for our visual experience. When edges are experimentally blurred (Troxler, 1804; Poletti & Rucci, 2010), masked (Paradiso & Nakayama, 1991; Salmela & Laurinen, 2009; Betz, Shapley, Wichmann, & Maertens, 2015a), or rendered invisible via adaptation (Anstis, 2013; Betz, Shapley, Wichmann, & Maertens, 2015b), human observers fail to segment objects from their background. Thus, edges are important features that support object segmentation (e.g., Morgan, 2011) and also play a relevant role in machine vision algorithms (e.g., Ferrari, Fevrier, Jurie, & Schmid, 2007; Zitnick & Dollár, 2014).

Over the past 50+ years, most research on contrast sensitivity has focused on smoothly varying periodic patterns such as sinusoidal gratings or Gabor patches, because they are well-defined in frequency space. According to the standard model of spatial vision (e.g., Graham, 2011), the visual system is composed of multiple spatial frequency (SF) selective channels, which can be individually probed with sinusoidal patterns. Another advantage of sinusoidal stimuli is that their contrast can be reduced to very low levels. This allows to characterize the visual system’s sensitivity at its limit and supposedly probe individual SF-selective channels with very little noise (Pelli & Farell, 1999).

However, smoothly varying stimuli have little resemblance to edges as we encounter them in the real world, and hence it has been questioned how much we can learn about edge sensitivity from experiments of that type. Georgeson, May, Freeman, and Hesse (2007), for example, express this concern: “It remains unclear how the output of this multiscale population of cells or filters is used to locate and describe the key features (*edges*) in images, and despite much progress there is no adequate standard model of feature analysis for human vision.” We also observed that variations of the standard spatial vision model cannot fully account for human edge sensitivity when exposed to a variety of noise patterns (Schmittwilken, Wichmann, & Maertens, 2024). Thus, the mechanisms underlying edge sensitivity are not yet fully understood (Carandini et al., 2005; Olshausen & Field, 2005).

**Figure 1.**
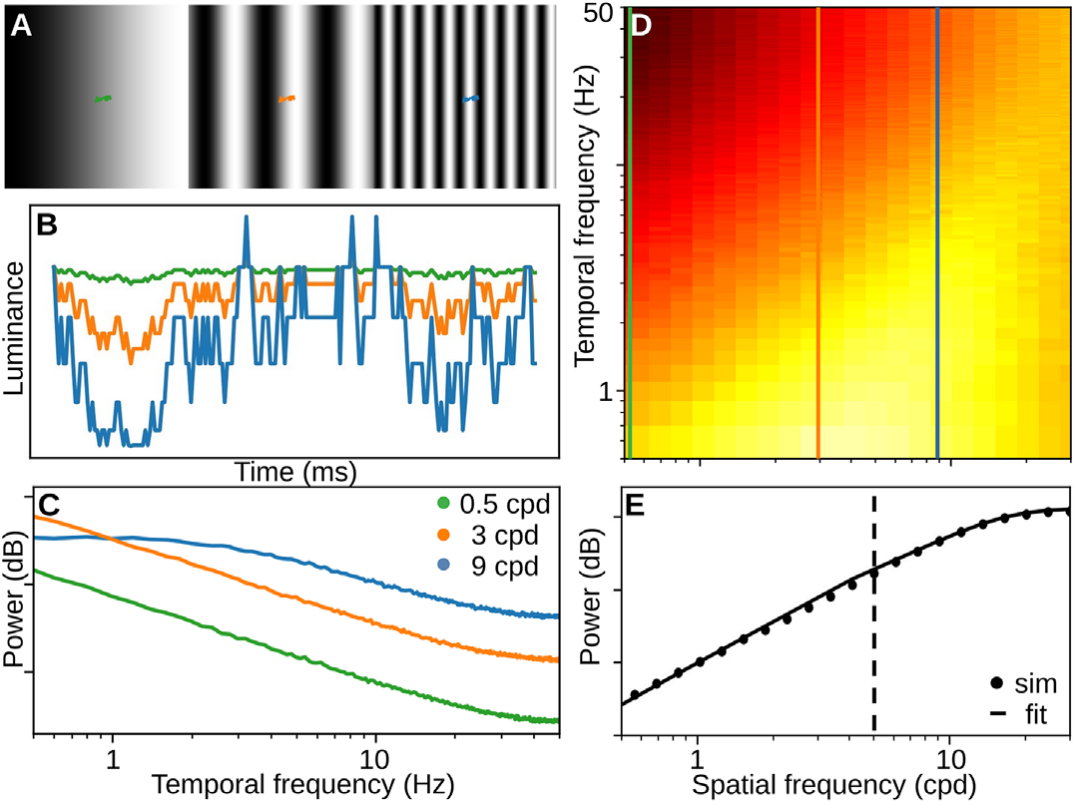
SF weighting due to ocular drift. (B) Luminance modulations that result from the drift trace (colored curves) on the gratings (0.5, 3, 9 cpd) in A. Drift modulations are stronger for high SFs. (C) Temporal power of signals in B over many instances. Drift increases temporal power for high SF inputs. (D) Same as C (vertical lines), but for a wider range of SFs. (E) If we average power in D across temporal frequencies (y-axis), we see how drift redistributes spatial power in the visual input. We fit this *drift gain* with Equation 1. Exact values were omitted because they change with signal contrast.

The standard model of spatial vision treats the visual input as *stationary*, at least for the duration of a fixation. However, in natural vision, involuntary eye movements, including microsaccades and smaller ocular drifts, continuously alter the visual input during fixations (Ratliff & Riggs, 1950; Ditchburn & Ginsborg, 1953) and modify its power distribution across different SFs (Figure 1; Rucci & Victor, 2015; Mostofi et al., 2020). As the eye drifts, individual receptors are stimulated by slightly different portions of the visual field over time (Figure 1A). These temporal variations are smaller for spatially homogeneous inputs than spatially variegated inputs (Figures 1A, 1B). The temporal power of the input thus critically depends on its spatial structure (Figure 1C). As a result, ocular drift dynamically redistributes power in the visual input, emphasizing high SF contents (Figure 1D; Kuang, Poletti, Victor, & Rucci, 2012). This SF-specific redistribution is inversely proportional to the power spectrum of natural scenes, effectively whitening their $\frac{1}{SF}$ content up until about 5 cpd (Figure 1E).

We propose that integrating ocular drift into our understanding of spatial vision offers a more accurate model of how the brain processes key features of natural scenes. In the present study, we explore how ocular drift contributes to edge sensitivity. Our analysis uses our recent empirical data (Schmittwilken et al., 2024), where we probed human edge sensitivity with 2D noise patterns. A spatial vision model with standard components was able to predict edge sensitivity in many but not all experimental conditions. Deviations emerged particularly for low SF edges and low SF noise. We quantify how ocular drift affects the predictions of a heuristic and a mechanistic model in response to the stimuli used in Schmittwilken et al. (2024). To anticipate, drift-induced changes to the power spectrum of the input improve the predictions of edge sensitivity in the heuristic test. We do not observe this in the responses of the mechanistic model. This discrepancy might result from current implementations of known mechanisms, which have been established with static inputs. We discuss how this may lead to bias and self-confirmatory theories.

## Methods

The code and data to reproduce the results can be found at https://github.com/computational-psychology/schmittwilken2025_edge-comparison.

### Edge sensitivity dataset

The empirical basis for the present investigation is our recent data on Cornsweet edges. We measured sensitivity to Cornsweet edges with three peak SFs (0.5, 3, 9 cpd) that were masked by 2D broadband (white, pink, brown) and narrowband noise (center SFs: 0.5, 3, 9 cpd; Schmittwilken et al., 2024). Variations of the standard spatial vision model predicted empirical sensitivity in many but not all tested conditions. We briefly describe the most relevant aspects of the empirical dataset here. A more detailed description is provided in Schmittwilken et al. (2024).

#### Stimuli

**Figure 2.**
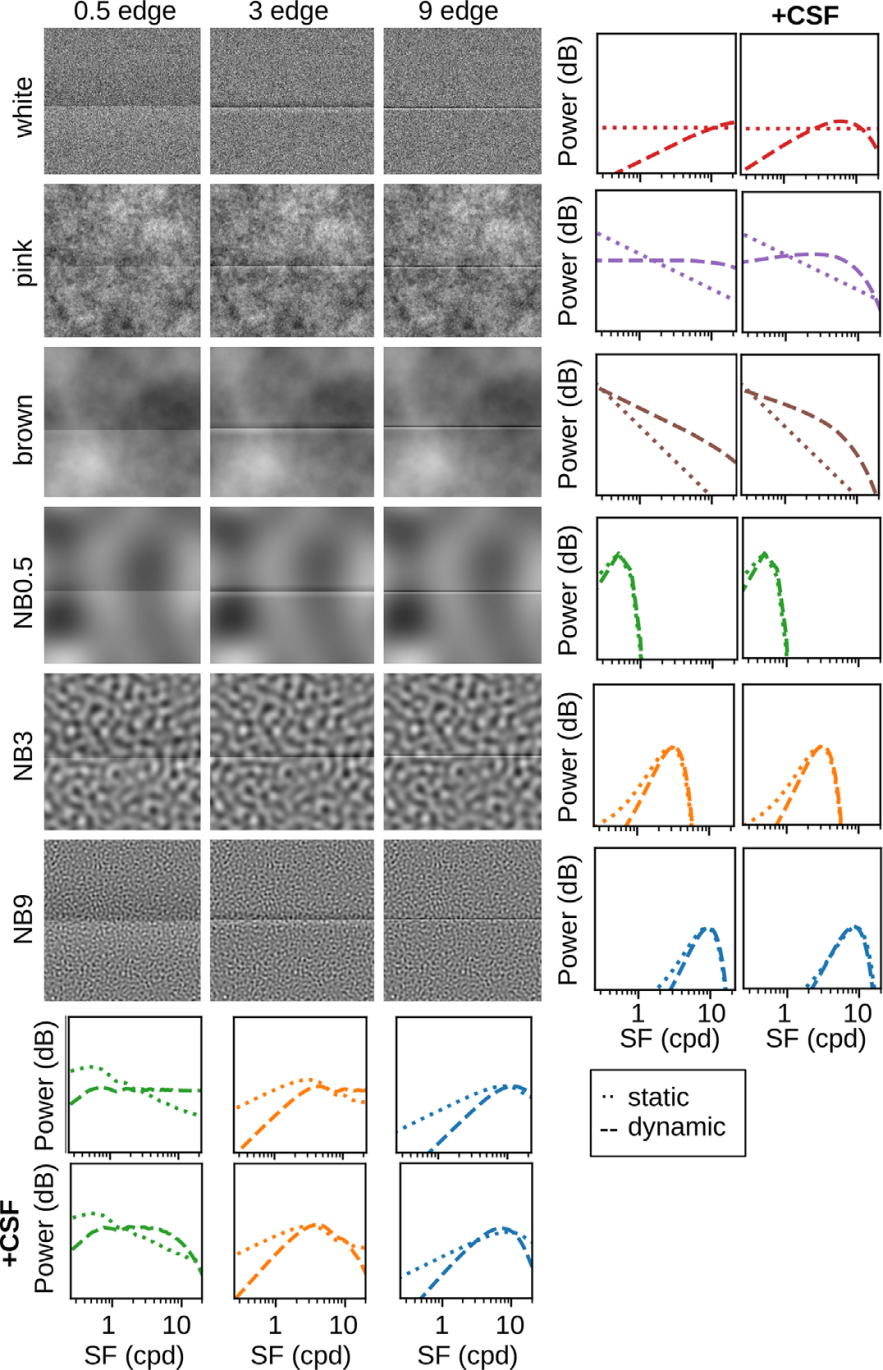
Stimuli from Schmittwilken et al. (2024) as well as their power spectra for stationary (*P*_*e*_ and *P*_*n*_; dotted curves) and dynamic (*P*_*e*_ · *D*, *P*_*n*_ · *D*; dashed curves) inputs. *+CSF* indicates the same power spectra weighted by the contrast sensitivity function. Drift shifts power toward high SFs, which whitens the $\frac{1}{SF}$ statistics of natural scenes, which is most visible for pink noise.

We used Cornsweet edges at three SFs and six types of noise with varying SF properties. All stimulus conditions except for no-noise and their SF spectra are shown in Figure 2. All stimuli were created with *stimupy 1.1* (Schmittwilken, Maertens, & Vincent, 2023).

Cornsweet edges are defined by a central luminance discontinuity, which linearly ramps into mean luminance on both sides of the edge. Ramp widths were 2.88 arcmin, 9 arcmin, and 58.8 arcmin, resulting in peak SFs of 9 cpd, 3 cpd, and 0.5 cpd. We tested five edge contrasts for each stimulus condition.

Each stimulus subtended 4 × 4° visual angle and contained a single horizontal edge that was placed 0.5° above or below its midline. Edge polarity was randomized. Mean luminance was 100 cd/m^2^.

For the noise masks, we used one of three broadband noises (white, pink, brown) and three narrowband noises (center SFs: 0.5, 3, 9 cpd) with one octave bandwidth. Noise root mean square (RMS) contrast was constant at 0.2, so that all noises had the same contrast energy (i.e., mean power).

#### Experimental design

Stimuli were shown on a linearized 21-inch Siemens SMM 21 106 LS CRT monitor (40 × 30 cm, 1,024 × 768 px, 130 Hz), controlled by a DataPixx toolbox (Vpixx Technologies, Inc., Saint-Bruno, QC, Canada). Observers (*N* = 6) maintained fixation in the center of the screen. We controlled viewing distance (100 cm) with a chin rest. Pixel resolution was 44 pixels/°.

We tested edge sensitivity in a spatial two-alternative forced-choice (2-AFC) task. Observers indicated whether the edge was above or below the stimulus midline. In each trial, the stimulus was faded in over a Hanning window (100 ms), displayed at full contrast (200 ms), and then faded out. A temporal frequency of 2.5 Hz most closely matches these temporal dynamics.

The experiment consisted of 42 blocks (two blocks per stimulus condition) in random order across two sessions. Overall, we collected 200 trials per stimulus condition spread over five edge contrasts. For each of those conditions, we fitted psychometric functions with *psignifit 4*, a Bayesian psychometric function estimation software package (Schütt, Harmeling, Macke, & Wichmann, 2016).

### Heuristic approach

We hypothesized that incorporating the consequences of ocular drift, and hence a *dynamic visual input*, could bridge the gap between the empirical edge sensitivity patterns and the model predictions in Schmittwilken et al. (2024). The heuristic test is based on the concept of channel interference within the standard spatial vision model. When stimuli and noise share similar spectral properties, they are processed within the same SF selective channels. This overlap results in a low signal-to-noise ratio and, consequently, reduced visual sensitivity (Pelli & Farell, 1999).

#### Drift gain

Drift was modeled as a 2D Brownian motion process with a diffusion coefficient of *D* = 20 $\frac{arcmin^{2}}{s}$ (Kuang et al., 2012). To understand how drift redistributes spatial power, we simulated its consequences on the retinal input (Figure 1). We fitted a function that captures the relationship between power and SF, and call this drift gain *D*(*f*). It allows us to emulate the effect of drift on the visual input for any stimulus, independent of its size and resolution.

(1) $$D\left(f\right)=\left\{\begin{array}{cc} f^{2}/f_{s}, & \text{if}f\leq 5\;\text{cpd},\\ A+\frac{K-A}{1+Qe^{-bf}}, & \text{if}f>5\;\text{cpd}, \end{array}\right.$$

where *f* is spatial frequency, and *f*_*s*_ = 178.15 for a smooth transition to the logarithmic part of the function. *A* = −0.13, *K* = 1.10, *Q* = 10.54, and *b* = 0.22 were fit to best capture the data in Figure 1E.

#### Channel interference

To predict empirical thresholds, we first quantify channel interference by pointwise multiplication of the stimulus and noise spectra (Figure 2, dotted curves). We then normalize the sum of this product to match the range of the empirical thresholds. When the signal and noise share substantial power in the same SF bands, the resulting product, and hence predicted thresholds, will be large. Conversely, if their power distributions do not overlap, the predicted thresholds will be smaller.

Before computing the overlap between stimulus and noise spectra, we weight their SF spectra with the human contrast sensitivity function (CSF at 2.5 Hz, as this most closely matched the temporal dynamics of stimulus presentation; Kelly, 1979) to account for the visual system’s differential sensitivity to different SFs. The CSF-weighted power spectra are also shown in Figure 2 (*+*CSF**).^1^

Schematically, the predicted thresholds for static inputs are given by

(2) $$\begin{array}{c} T_{static}=norm\left\{\sum (P_{e}\cdot CSF\cdot P_{n}\cdot CSF)\right\},\; \end{array}$$

where *T*_*static*_ represents the predicted threshold, *P*_*e*_ is the power spectrum of the edge stimulus, and *P*_*n*_ is the power spectrum of the noise pattern.

To model the effect of drift on the visual input, we multiply the edge and noise spectra by the drift gain *D* (Figure 1E, Equation 1). The dynamic spectra exhibit less power at low SFs and more power at high SFs compared to the static spectra (Figure 2, dashed curves). The predicted thresholds for dynamic inputs are then given by

(3) $$\begin{array}{c} \;T_{dynamic}=norm\left\{\sum (P_{e}\cdot CSF\cdot D\cdot P_{n}\cdot CSF\cdot D)\right\}\; \end{array}$$

Finally, we compare the empirical thresholds with the predicted thresholds for the static and dynamic visual inputs.

### Mechanistic approach

**Figure 3.**
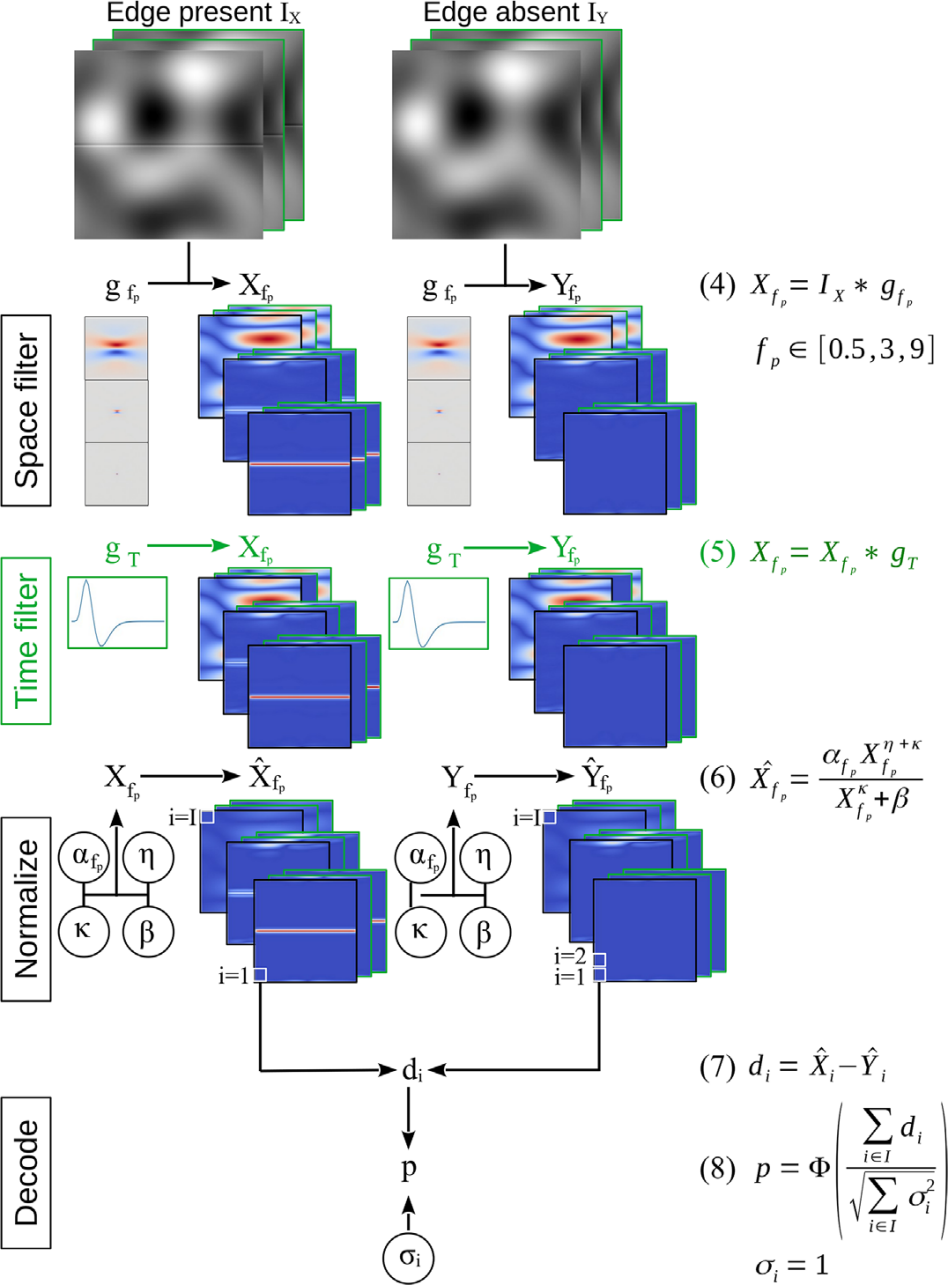
Model overviews. Extensions of the active model are highlighted in green. The models take two inputs, edge present *I*_*X*_ and edge absent *I*_*Y*_, and apply linear filters, a nonlinear normalization, and a *d*′-decoder to compare the model outputs with the empirical data. The filters $g_{f_{p}}$ and *g*_*T*_ are defined in Equations 9–13. All spatial filters have the same shape and only vary in scale. The six normalization parameters were fitted to the empirical data. $\alpha_{f_{p}}$ weights each SF channel. β, κ, and η determine the shape of the Naka–Rushton function.

In the second step, we investigate the effect of ocular drift on edge sensitivity in a mechanistic model. For this, we extended the best-performing variant of the standard model of spatial vision from Schmittwilken et al. (2024) by a temporal dimension (green markings in Figure 3). Both the original spatial model and the novel active model share key components of early visual processes: linear filtering with three log-Gabor filters tuned to the peak SFs of the edge stimuli (0.5, 3, 9 cpd), nonlinear normalization, and a decoder rooted in signal detection theory (Heeger, 1992; Schütt & Wichmann, 2017). Specifically, we use a Naka–Rushton function to implement normalization localized in space, SF, and time, along with a *d*′-decoder for perceptual decision-making.

All filter parameters were derived from psychophysical and neurophysiological studies (for details, see Schmittwilken et al., 2024). In total, we fitted six normalization parameters to the empirical data: three $\alpha_{f_{p}}$ parameters, which modulate the contribution of each SF channel to the model output, and three parameters that control the shape of the nonlinear normalization (β, κ, and η). In the following, we briefly describe all model components step by step.

#### Model input

To emulate the 2-AFC nature of the task, we always present two inputs to the models, one containing the signal plus noise (edge-present, *I*_*X*_) and one containing only noise (*I*_*Y*_). For the spatial model, *I*_*X*_ and *I*_*Y*_ are images. For the active model, *I*_*X*_ and *I*_*Y*_ are series of images that mimic the dynamic sampling strategy of the visual system via ocular drift. Based on empirically observed drift, we simulate individual drift instances as 2D Brownian motion with a diffusion coefficient of *D* = 20 $\frac{arcmin^{2}}{s}$ (Kuang et al., 2012). We simulate each instance over a time period of *T* = 200 ms with a sampling frequency of *f*_*T*_ = 200 Hz.

#### Spatial filters

We use odd-symmetric log-Gabor filters because they emulate properties of cells in the early visual pathway (Morrone & Burr, 1988; Schütt & Wichmann, 2017) and optimally respond to step edges (Shapley & Tolhurst, 1973; Morrone & Burr, 1988). The filters $g_{f_{p}}$ are defined as an imaginary part of the inverse Fourier transform of $G_{f_{p}}$.

(9) $$\begin{array}{c} g_{f_{p}}\left(x,y\right)=Im\left[F^{-1}\left\{G_{f_{p}}\left(f,\theta \right)\right\}\right]\; \end{array}$$

(10) $$\begin{array}{c} G_{f_{p}}\left(f,\theta \right)=exp\left(\frac{-log{\left(f/f_{p}\right)}^{2}}{2log{\left(\sigma_{f_{p}}\right)}^{2}}+\frac{-\theta^{2}}{2\sigma_{\theta }^{2}}\right),\; \end{array}$$

where *f* represents spatial frequency (cpd) and θ the absolute angular distance from the orientation of the edge (°). The filters’ peak SFs were set to *f*_*p*_ = [0.5, 3, 9] cpd, corresponding to the peak SFs of the edges. We set the SF bandwidth to $\sigma_{f_{p}}=0.5945$ (1.4 octaves; no unit) and the orientation bandwidth to σ_θ_ = 0.2965 (20° half bandwidth; no unit) based on empirical data (Campbell & Kulikowski, 1966; Blakemore & Campbell, 1969; Ringach, Shapley, & Hawken, 2002).

#### Temporal filter

In addition to filtering in space, the active model also filters the visual input in time, emulating the temporal properties of the visual system. For this, we use a bandpass filter *g*_*T*_ (Supplementary Figure S2), which was fitted to the temporal contrast sensitivity data of (Robson, 1966):

(11) $$\;g_{T}\left(t\right)=\xi \left[\;h_{1}\left(t\right)-\zeta h_{2}\left(t\right)\;\right]\;1/f_{T}$$

(12) $$\;h_{1}\left(t\right)={\left(t/\tau \right)}^{n_{1}-1}\;exp\left(-t/\tau \right)\;/\;\left(\tau \left(n_{1}-1\right)!\right)$$

(13) $$\;h_{2}\left(t\right)={\left(t/\tau k\right)}^{n_{2}-1}\;exp\left(-t/\tau k\right)\;/\left(\tau k\left(n_{2}-1\right)!\right)$$

where *t* represents time (seconds), *k* = 1.33, *n*_1_ = 9, *n*_2_ = 10, $\tau =\frac{4.3}{1000}$, ζ = 1, ξ = 269 (Watson, 1986).^2^

#### Normalization

After filtering the visual input at each spatial (and temporal) position, we compute the absolute filter outputs to remove sensitivity to edge polarity (i.e., we perform full-wave rectification).

We then emulate nonlinearities in the early visual system (Legge & Foley, 1980) with a Naka–Rushton function (Naka & Rushton, 1966; Equation 6 in Figure 3). Other than the filter parameters, which were determined based on our experimental design and the literature, we fitted the six Naka–Rushton parameters to best predict the empirical data.

#### Decoding

To derive model performance, we computed *d*′ between the multidimensional model outputs for *I*_*X*_ and *I*_*Y*_ (Equations 7–8 in Figure 3). We set the internal noise to unit-variance instead of fitting it to avoid covariation with $\alpha_{f_{p}}$ in the Naka–Rushton function (as demonstrated in Schmittwilken et al., 2024).

#### Parameter fitting

We used the same maximum likelihood approach to separately fit the six Naka–Rushton parameters of the spatial and active model to the empirical data, as in Schmittwilken et al. (2024). The approach consisted of a manual grid search followed by an automatic optimization with a Simplex search algorithm. The initial parameter ranges of the grid search were guessed based on Schmittwilken et al. (2024).

For each parameter combination, we generated model predictions in all stimulus conditions (5 contrasts, 3 edges, 7 noises = 105 data points). To avoid that single noise or drift instances biased model performances, we averaged model predictions for each data point over *N* = 30 repetitions. Finally, we summed the log-likelihoods of the model predictions given the empirical data. The final model parameters are hence the result of maximizing this log-likelihood across all data points.

## Results

### Heuristic test of the effect of drift

**Figure 4.**
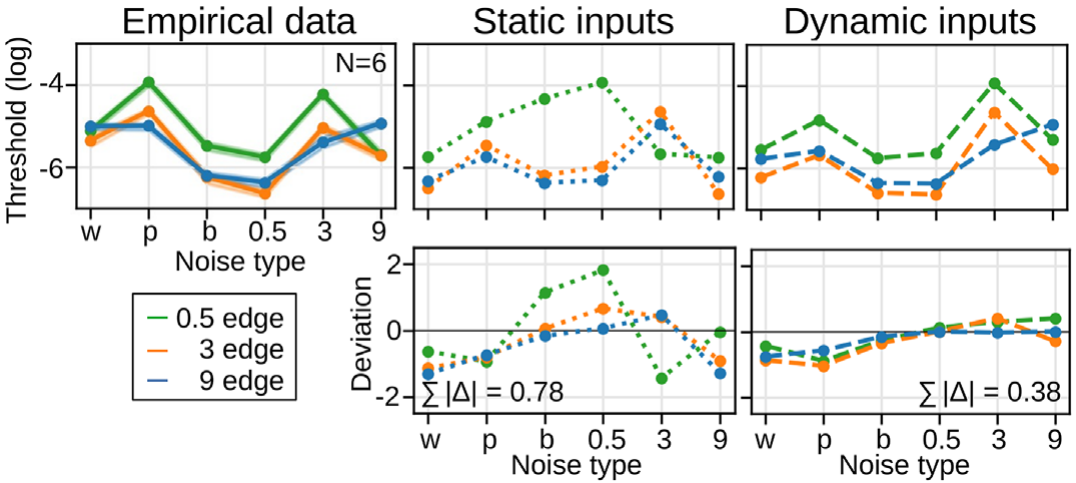
Effect of drift on the relationship of signal and noise. Empirical and predicted thresholds (top row), and the difference between empirical and predicted thresholds (bottom row), for all combinations of edge and noise conditions. Shaded areas for the empirical data represent 68% credible intervals. Values for the static and dynamic inputs are fully deterministic.

Figure 4 compares the empirical data (75%-thresholds) with the predicted thresholds for both static and dynamic inputs. The predictions for dynamic inputs are closer to the empirical data than those for static inputs. The difference between predicted and empirical thresholds is approximately half as large for dynamic inputs ($\Sigma |\bar{\Delta }|$ = 0.38) as for static inputs ($\Sigma |\bar{\Delta }|$ = 0.78). This suggests that ocular drift alters the stimulus power such that it better predicts human edge sensitivity, without incorporating additional mechanisms into the model or fitting any parameters.

**Figure 5.**
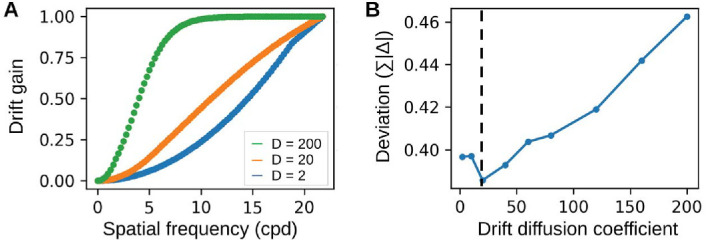
Dependence on drift magnitude. (A) Faster drifts preferentially amplify lower SFs, while slower drifts emphasize higher SFs. The empirically observed diffusion coefficient is $D=20\frac{arcmin^{2}}{s}$ (Kuang et al., 2012). (B) Model predictions are most accurate at *D* = 20, where the discrepancy between empirical and predicted thresholds is minimal.

To investigate how predictions for the dynamic inputs change with drift magnitude, we repeated the analysis and varied the diffusion coefficient *D*. Changes in drift magnitude influence the drift gain, that is, the extent to which drift redistributes stimulus power across SF bands (Figure 5A). Larger drift, for example (green curve in Figure 5A), amplifies information at lower spatial frequencies. To quantify how model predictions change with different drift diffusion coefficients, we calculate the same deviation measure ($\Sigma |\bar{\Delta }|$) as above. Predictions were most accurate for empirically observed drift magnitudes (*D* = 20) and deteriorated for smaller and larger values of drift (Figure 5B).

### Effect of drift in a multiscale model

The similarity relationship between signal and noise in the dynamic inputs does not fully account for the empirical data, as it underestimates the effect of white (w) and pink (p) noise on edge visibility. To address this, we also simulate the effect of drift within a mechanistic model.

**Figure 6.**
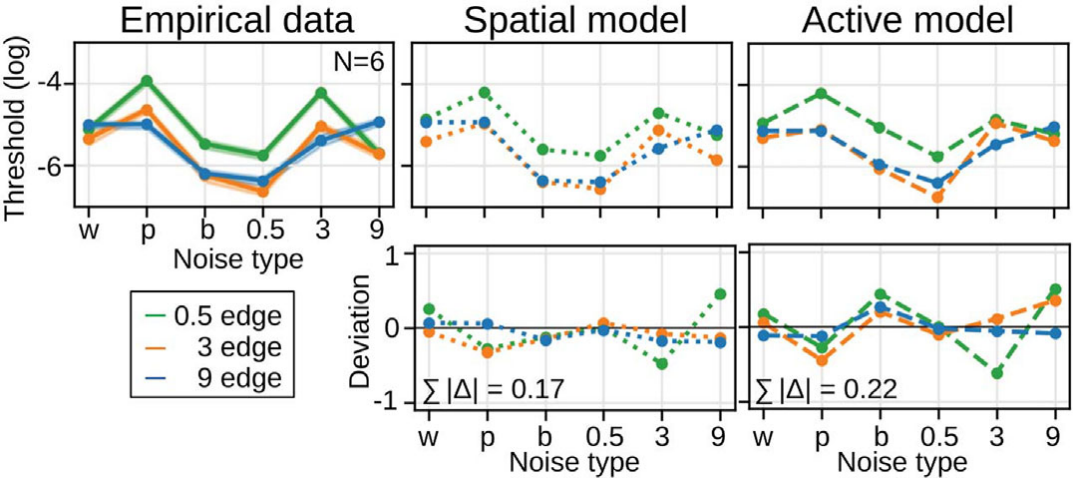
Empirical and multiscale model thresholds for the spatial and active models (75% performance, top row), and the difference between empirical and model thresholds (bottom row) for all edge and noise conditions. Shaded areas for the empirical data represent 68% credible intervals.

We conducted separate fits for the spatial and active models, and assessed goodness of fit as mean squared deviance residuals between model predictions and individual observers’ data (Collett, 2002; Schmittwilken et al., 2024).^3^ Both models were able to predict human edge sensitivity in many stimulus conditions, though not all (Figure 6; Supplementary Figure S4 for full psychometric curves). Notably, the spatial model outperformed the active model, yielding a deviance per data point of 2.6 compared to 3.1, respectively. This difference was particularly pronounced for the low SF edge in the presence of brown (b) and 3 cpd (3) noise.

Next, we explored why the spatial model outperformed the active model. This seemed inconsistent with the beneficial effects of ocular drift in the heuristic test. A comparison of the fitted parameters (Table 1) revealed substantial differences in $\alpha_{f_{p}}$ parameters between the spatial and the active model. They determine how much each SF channel contributes to the model output. The spatial model places an emphasis on the high SF channel relative to the low SF channel (α_9_ = 21 * α_0.5_), while the active model weights the two channels more evenly, with a slight emphasis on the low SF channel (α_0.5_ = 3.7 * α_9_).^4^

**Table 1.** Fitted normalization parameters for the spatial and active model. We show the raw parameters, as well as the relative contribution of each SF channel to the model output, reflected in the α-parameters. The spatial model drastically shifts power toward higher SFs in the model output, similar to the effect of ocular drift in the model input. The contribution of SF channels in the active model is more balanced.

	Spatial model		Active model	
α_0.5_	0.063	1.78%	3.093	37.93%
α_3_	2.172	61.49%	4.234	51.93%
α_9_	1.297	36.72%	0.827	10.14%

β	0.649		2.6e-16	
η	0.301		1.126	
κ	1.774		6.631	

Both models transform the spatial frequency spectrum such that high SFs get a relatively stronger weight. The active model redistributes stimulus power in the model input, whereas the spatial model reweights SF contents in the model output. The spatial model thus shifts SF information in a manner similar to the effect introduced by ocular drift. Using this SF-specific weighting, the spatial model outperformed the active model.

### Effect of drift in a single-scale model

A central assumption of pattern vision is that spatial vision operates through narrowly tuned SF channels (Graham, 2011, for review). In line with this conception, we have demonstrated recently that a spatial vision model with a single channel cannot account for human edge sensitivity (Schmittwilken et al., 2024). Our current study suggests that the spatial model requires the existence of multiple narrow channels in order to reweight the contribution of the spatial frequency channels similar to ocular drift. This raises the question whether the existence of these channels is still necessary once we consider the effect of ocular drift on the visual input.

To test this, we implemented a version of the spatial and active models, in which we replaced the narrowly tuned log-Gabor filters with a single, broad log-Gabor filter, which was fitted to the contrast sensitivity function at 2.5 Hz (Kelly, 1979).

**Figure 7.**
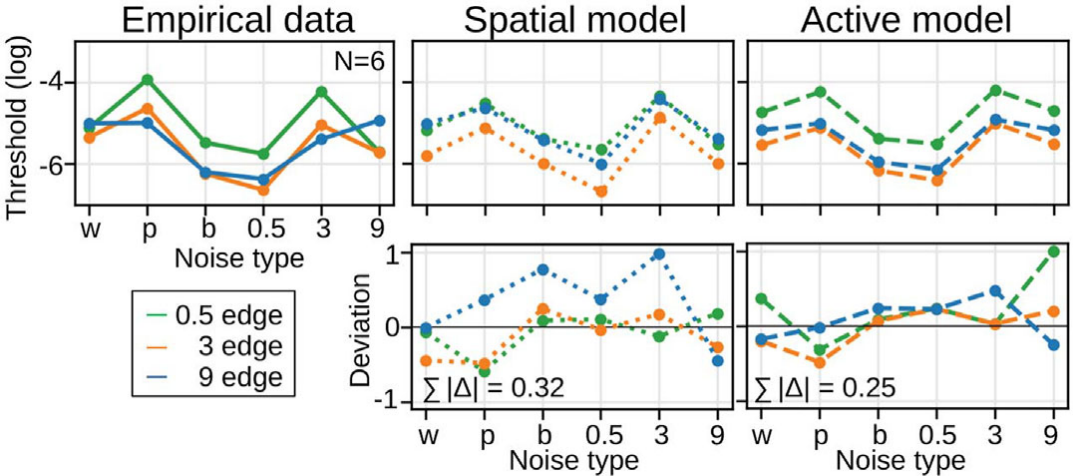
Empirical and single-scale model thresholds for the spatial and active models (75% performance, top row) and the difference between empirical and model thresholds (bottom row) for all edge and noise conditions. Shaded areas for the empirical data represent 68% credible intervals.

Figure 7 shows the thresholds of the single-scale models. We first focus on the behavior of the spatial models. As expected, the predictions of the spatial single-scale model deviate more strongly from the empirical data than the predictions of its multiscale version ($|\bar{\Delta }|$ = 0.32 compared to $|\bar{\Delta }|$ = 0.17 in Figure 6). In contrast, the predictions of the drift-enhanced models are more similar ($|\bar{\Delta }|$ = 0.25 compared to $|\bar{\Delta }|$ = 0.22 in Figure 6). This is a first indication that human edge processing does not require the existence of multiple narrow channels if we acknowledge the effect of fixational eye movements for visual processing, though the effect was more variable between noise instances in the single-scale models.

## Discussion

Pattern vision has traditionally been studied as a *spatial* process (Graham, 2011, for review). We move our eyes across the visual scene and fixate on different points of interest. At each of those fixation locations, we acquire a snapshot of the scene (Rucci, Ahissar, & Burr, 2018). This camera analogy implicitly assumes that the retinal input during a fixation can be characterized as a static image, and hence subsequent visual processes are spatial in nature. Historically, we see how the camera analogy has affected both, how we describe and how we study the visual system. Many fundamental insights, such as the receptive field properties of cells in the early visual pathway or their interactions, have been derived from neurophysiological data with anesthetized (i.e., immobilized) animals or an eye in a dish (Hubel & Wiesel, 1968; Maffei & Fiorentini, 1973; Movshon, Thompson, & Tolhurst, 1978; Kaplan & Shapley, 1986; Croner & Kaplan, 1995; Carandini, Heeger, & Movshon, 1997; Kohn & Smith, 2005).

The camera analogy has been instrumental for our current understanding of visual processing, but it overlooks two of its key aspects:

(1) The visual system is highly sensitive to temporal changes, as much so that vision deteriorates in the absence of visual transients (Robson, 1966; Kelly, 1979). The phenomenon of visual fading, first described by Troxler (1804), emphasizes the importance of temporal stimulus modulations.
(2) Even during fixations, the eyes are never truly at rest. We perform involuntary, microscopic eye movements, such as ballistic microsaccades, every ∼250 ms, and slower, erratic ocular drifts that continuously occur in between (Ratliff & Riggs, 1950; Ditchburn & Ginsborg, 1953).

Recent advances in eye tracking and display technologies revealed that these microscopic eye movements constitute an active sampling strategy of the visual system to process information in space and time (Martinez-Conde, Macknik, Troncoso, & Dyar, 2006; Rucci & Victor, 2015; Witten, Lukyanova, & Harmening, 2024). In particular, ocular drift has been shown to play a role in contrast sensitivity (Boi, Poletti, Victor, & Rucci, 2017; Casile, Victor, & Rucci, 2019) and visual acuity (Ratnam, Domdei, Harmening, & Roorda, 2017; Intoy & Rucci, 2020; Nghiem, Witten, Dufour, Harmening, & Azeredo da Silveira, 2025) and may contribute to edge extraction (Schmittwilken & Maertens, 2022). These findings suggest that the retinal input is better characterized as a spatiotemporal flow, which is determined by the spatial structure of the visual scene and the temporal dynamics of eye movements (Figure 1).

Ocular drift shifts the spectral power of the input toward higher SFs (Figure 1; Kuang et al., 2012; Rucci & Victor, 2015). We hypothesized that incorporating this effect in a standard spatial vision model could improve the model’s predictions for edge sensitivity in noise (Schmittwilken & Maertens, 2022). We tested this hypothesis in two ways. In the heuristic test, we quantified interference between edge and noise spectra for both static and dynamic inputs, the latter incorporating the effect of drift. Predictions based on the dynamic inputs captured the empirical data more accurately than those based on static inputs, but they did not fully account for the results (Figure 4). Furthermore, we found that the dynamic predictions were optimal for empirically observed drift, with performance deteriorating for both smaller and larger values (Figure 5). This underscores the delicate interplay between fixational eye movements and visual sensitivity (Intoy et al., 2024), though dynamic predictions generally remained better than those based on static inputs across a broad range of drift magnitude (*D* = [2; 200]).

Second, we extended a mechanistic standard spatial vision model (Schmittwilken et al., 2024) by a temporal filter and tested its predictions to a dynamically sampled input (Figure 3). The results confirmed that emphasizing high SFs, which results from sampling via drift, leads to a more accurate prediction of human edge sensitivity. However, a spatial model optimized in the absence of drift uses parameters at the output stage (Table 1), which “emulate” the effect of drift by giving a higher weight to the high SF selective channel. Using this SF channel selective weighting scheme, the spatial model outperformed the active model (Figure 6). To reiterate, both models give more weight to high SFs. In the active model, this happens via drift-induced sampling at the input stage. In the spatial model, this happens via channel-specific weighting at the output stage. Given that most model architectures and parametrizations have evolved and been optimized to account for static inputs (Graham, 2011, for review), it is difficult to establish a fair comparison between active and static models of processing, because data (dynamic vs. stationary) and theory (active vs. static) stimulate and reinforce each other (cf. Carandini et al., 2005; Olshausen & Field, 2005).

A factor that might contribute to the spatial model's superior performance is the choice of the spatial filter. Log-Gabor filters are designed to reflect the receptive field properties of cells in the early visual system (Movshon, Thompson, & Tolhurst, 1978; Morrone & Burr, 1988; Schütt & Wichmann, 2017). However, as outlined above, their exact parametrization is based on empirical data influenced by the camera analogy, that is, neurophysiological data with immobilized eyes (e.g., Hubel & Wiesel, 1968; Movshon, Thompson, & Tolhurst, 1978). Considering the implications of fixational eye movements may necessitate revisions to both the experimental design and analysis tools, such as reverse correlation or spike-triggered averages. These might substantially change our definition of the spatial filters.

We have previously shown that, when ocular drift is considered, an active early vision model robustly detected edges in the absence of orientation-selective processes (Schmittwilken & Maertens, 2022). Temporal filtering of an actively sampled input converted its temporal variations into discontinuities in space (i.e., edges). This finding invites reconsideration of the interpretation of orientation selective cells in V1 as edge detectors (cf. Prokopowicz & Cooper, 1995).

Another example is the assumption that spatial vision operates through narrowly tuned SF channels. The spatial model redistributes stimulus power toward higher SFs by a corresponding reweighting of individual SF channel outputs. This compensation via differential channel weighting is possible, because the model presupposes the existence of such narrowly tuned channels. If, instead, edge processing is mediated by a broader SF channel, then redistributing power across individual channels would not be feasible. This raises an important question: Does human edge processing require multiple, narrowly tuned SF channels, or is a single, broader SF channel sufficient? The debate is currently unresolved (e.g., Elder & Sachs, 2004; McIlhagga, 2018), and the effect of fixational eye movements on different models has not yet been tested. To test this, we implemented a version of the spatial and active models, in which we replaced the narrowly tuned log-Gabor filters with a single, broad log-Gabor filter, which was fitted to the contrast sensitivity function at 2.5 Hz (Kelly, 1979). As expected, the performance of the spatial single-scale model was much worse compared to the active single-scale model, which performed similarly to its multiscale version (Figure 7). This indicates that human edge processing indeed does not require the existence of multiple narrow channels, once we acknowledge the effect of fixational eye movements for visual processing.

Allowing for the possibility that edge processing may not require orientation or SF selective cells opens the possibility that edge extraction occurs prior to V1 to a larger degree than traditionally assumed. The idea that temporal response variations of cells at the front end of the visual system can be used for edge detection has been around for a while (e.g., the resonant retina; Prokopowicz & Cooper, 1995; Hongler, de Meneses, Beyeler, & Jacot, 2003). In robot vision, so-called event-based algorithms are employed that detect changes over time, efficiently coding for features such as edges (Gallego et al., 2020).

If edge detection is already occurring to a large degree before visual cortex, then the question arises once again what V1 is doing (Carandini et al., 2005; Olshausen & Field, 2005). Most likely, cells in V1 already respond to higher-level features of the visual input (Lee, Mumford, Romero, & Lamme, 1998; Olshausen & Field, 2005). Recent work showed that already at short latencies (∼50 ms), neurons in V1 do not respond to all edges alike but prefer perceptually relevant object boundaries (Papale et al., 2024). Thus, a paradigm shift from a purely spatial to a spatiotemporal view of visual processing could lead to a critical re-evaluation of long-standing assumptions of visual neuroscience that are based on the camera analogy.

While both the spatial and active models in this study have their merits, performance improvements can certainly be achieved by further optimizing the model components (filters, normalization schemes, etc.). We intentionally limited our models to standard components in order to contrast two fundamentally different perspectives on visual processing: the spatial view, where spatial information is encoded in retinotopic locations, and the active view, where spatial information is encoded through a spatiotemporally varying input stream (Rucci & Victor, 2015). We argue that fixational eye movements introduce jitter across the photoreceptor array, which likely carries information and constitutes an active sampling strategy, suggesting that a spatiotemporal input pattern is necessary for a more accurate mechanistic model of pattern vision.

In conclusion, while the exact mechanisms underlying pattern vision are still being explored, our findings suggest that we need to incorporate the dynamic nature of the visual input into our models. Future research should explore the implications of fixational eye movements and reconsider the nature of spatial filters involved in contrast and edge sensitivity. This could provide valuable insights into the dynamics of visual processing and how early visual mechanisms facilitate higher-level perceptual processes.

## Supplementary Material

Supplement 1

## Footnotes

1 Substituting the CSF model by a more recent version (Ashraf, Mantiuk, Chapiro, & Wuerger, 2024) only marginally affected our results. Interestingly, however, any changes to the “correct” CSF (e.g., by varying the temporal frequency of the CSF away from the temporal dynamics of stimulus presentation) worsens the predictions for both the static and dynamic inputs. This underpins how the effect of drift perfectly interplays with the CSF to predict empirical sensitivity patterns. The spatial profiles of both CSF functions are shown in Supplementary Figure S1.

2 Substituting the temporal filter with alternatives, for example, based on the temporal dynamics of V1 simple cells (Zheng et al., 2007), retinal ganglion cells (Benardete & Kaplan, 1999), or other contrast sensitivity functions (Kelly, 1979), only marginally affected the results. This finding is in agreement with a previous observation that the exact properties of the temporal filter do not affect edge detection as long as it is sensitive to luminance transients over time (cf. Schmittwilken & Maertens, 2022). All temporal profiles are shown in Supplementary Figure S2.

3 Deviance residuals are analogous to the unit-variance, normally distributed deviations that constitute the χ^2^-statistic. The best possible deviance, given by the psychometric function fit, was 1.8.

4 Fitted Naka–Rushton functions can be found in Supplementary Figure S3.
