# Supplementary material for: Ocular drift shakes the stationary view on pattern vision
Source: J Vis. 2025 Jul 23;25(8):17. doi: 10.1167/jov.25.8.17 (PMC12306695; doi:10.1167/jov.25.8.17)
Supplement: Supplement 1 [file jovi-25-8-17_s001.pdf]

Supplement for "Ocular drift shakes the stationary view on pattern vision"

Lynn Schmittwilken and Marianne Maertens

1 Heuristic test: Contrast sensitivity function(s)

We used the CSF-model by Kelly (1979) at a temporal frequency of 2.5 Hz, because it most closely matched the temporal dynamics of stimulus presentation in the experiment. The CSF-profile is shown in Figure 1. In addition, we substituted the Kelly-CSF model by a more recent version called castleCSF (Ashraf, Mantiuk, Chapiro, & Wuerger, 2024). The two CSF-profiles are similar at low spatial frequencies but start diverging at high spatial frequencies. One reason for this divergence could be that the data that went into castleCSF in parts reflects the effect of ocular drift because not all sensitivity measurements were performed under retinal stabilization. As a consequence, the Kelly-CSF seems more suitable for our modeling approach. However, we also found that substituting the CSF model only marginally affected our results. We therefore only report the results with the Kelly-CSF model.

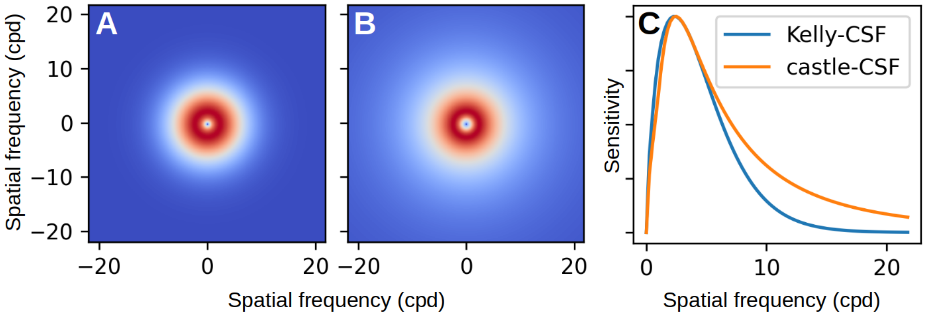

Supplementary Figure 1: Two-dimensional contrast sensitivity functions based on (A) Kelly, (B) castleCSF, and their one-dimensional profiles (C).

2 Mechanistic models

2.1 Spatial and temporal filters

Both mechanistic models employed three log-Gabor filters to emulate the spatial properties of early visual processes (Fig. 2A-D). The filters' center frequencies were matched to the peak frequencies of the edge stimuli, i.e. 0.5, 3 and 9 cpd. All other parameters were based on psychophysical and physiological data. In addition, the active model employed a temporal filter (Fig. 2E). For the main results, this filter was fitted to temporal contrast sensitivity data by Robson (1966) provided in Watson (1986). Alternative filters fitted to temporal sensitivity data of V1 (Zheng et al., 2007), retinal ganglion cells (Benardete & Kaplan, 1999), and alternative CSF models (Kelly, 1979) only marginally changed the results. This finding is in line with the observation that the exact properties of the temporal filter do not affect edge detection as long as it is sensitive to luminance transients over time (Schmittwilken & Maertens, 2022).

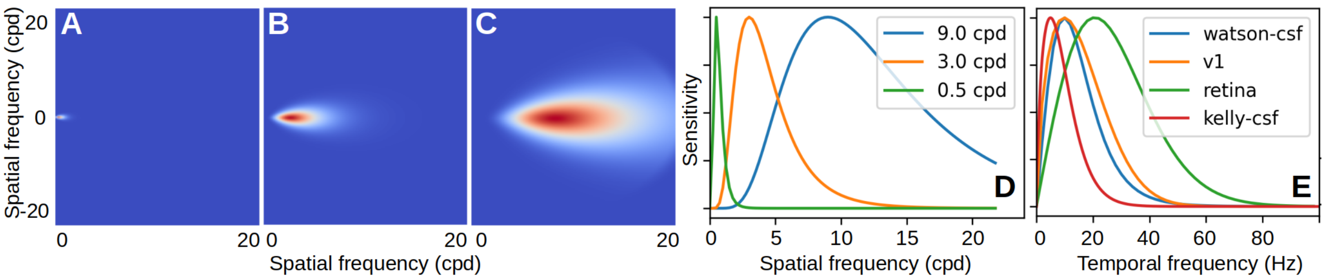

Supplementary Figure 2: Log-Gabor filters of the mechanistic models with center frequencies of (A) 0.5, (B) 3, and (C) 9 cpd, as well as their one-dimensional profile (D). (E) Sensitivity profiles of the temporal filter of the active model (blue curve) as well as the sensitive profiles of the filters that we used as substitution.

2.2 Fitted Naka-Rushton functions

The parameters of the Naka-Rushton functions were fitted to optimally predict the empirical data. Figure 3 shows the resulting Naka-Rushton functions for the spatial (A) and active model (B). They gray area shows the distribution of values that enter the Naka-Rushton functions.

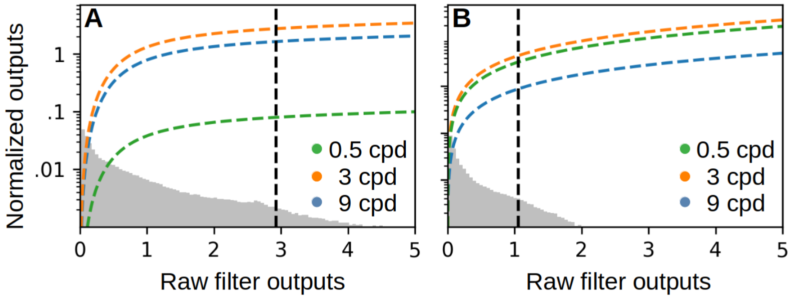

Supplementary Figure 3: Fitted normalization functions of the spatial (A) and active models (B). The colored graphs within one panel have the same general shape, but their differences represent how much each spatial-frequency-channel contributes to the model output (i.e. differences in α-parameters). The vertical dashed line indicates where 95% of the values lie that enter the normalization.

2.3 Model psychometric curves

Figure 4 shows the empirical edge sensitivity functions alongside the model psychometric curves for all edge and conditions.

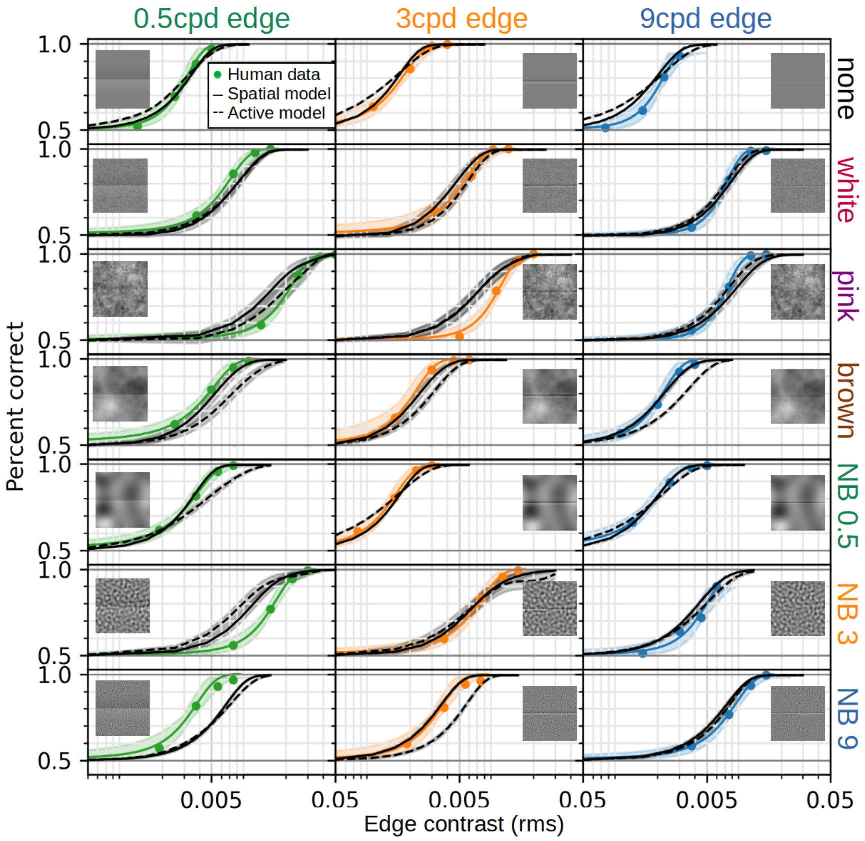

Supplementary Figure 4: **Model performances**. (A) Psychometric curves of spatial (continuous curves) and active model (dashed curves) alongside the empirical edge sensitivity functions (colored curves) for the 3 edges (columns) and 7 noises (rows). Icons represent example stimuli. Shaded areas show 68% credible intervals. NB: Narrowband.

References

Ashraf, M., Mantiuk, R. K., Chapiro, A., & Wuerger, S. (2024). castlecsf — a contrast sensitivity function of color, area, spatiotemporal frequency, luminance and eccentricity. *Journal of Vision*, *24*(4), 5–5. doi: 10.1167/jov.24.4.5

Benardete, E. A., & Kaplan, E. (1999). The dynamics of primate m retinal ganglion cells. *Visual neuroscience*, *16*(2), 355–368. doi: 10.1017/S0952523899162151

Kelly, D. H. (1979). Motion and vision. II. Stabilized spatio-temporal threshold surface. *JOSA*, *69*(10), 1340–1349. doi: 10.1364/JOSA.69.001340

Robson, J. G. (1966). Spatial and temporal contrast-sensitivity functions of the visual system. *JOSA*, *56*(8), 1141–1142. doi: 10.1364/JOSA.56.001141

Schmittwilken, L., & Maertens, M. (2022). Fixational eye movements enable robust edge detection. *Journal of Vision*, *22*(8), 5–5. doi: 10.1167/jov.22.8.5

Watson, A. B. (1986). Temporal sensitivity. In (Vol. 1, pp. 1–43). Wiley, New York.

Zheng, J., Zhang, B., Bi, H., Maruko, I., Watanabe, I., Nakatsuka, C., … Chino, Y. M. (2007). Development of temporal response properties and contrast sensitivity of V1 and V2 neurons in macaque monkeys. *Journal of Neurophysiology*, *97*(6), 3905–3916. doi: 10.1152/jn.01320.2006
